# Innovative Use and Characterization of Polymers for Timber-Related Construction

**DOI:** 10.3390/ma3021104

**Published:** 2010-02-10

**Authors:** Antony Darby, Tim Ibell, Mark Evernden

**Affiliations:** Department of Architecture and Civil Engineering, University of Bath, Bath, BA2 7AY, UK; E-Mails: T.J.Ibell@bath.ac.uk (T.I.); M.Evernden@bath.ac.uk (M.E.)

**Keywords:** fibre reinforced polymers, creep, timber, basalt, gridshells

## Abstract

Timber gridshells have become a very popular, efficient, sustainable and beautiful structural application of timber. However, given the slender laths involved in this form of construction, there is concern over the durability of timber for this purpose, and Glass FRP (GFRP) laths have been proposed as a possible substitution. This paper considers this possibility. It goes on to look at the possible use of Basalt FRP (BFRP) for the same purpose, from the perspective of its creep characteristics. It is shown that the use of GFRP gridshells is a viable form of construction, and that enhanced durability characteristics of BFRP could lead to their adoption for gridshells, given that the creep characteristics of basalt fibres presented here are comparable to those of glass fibres. An altogether different form of timber construction is that of joist-and-floorboard. In the UK, there are thousands of historic buildings which use this floor construction, and a sizeable proportion of this building stock now requires upgrade, strengthening and/or stiffening to allow these buildings to be fit for purpose into the future. This paper goes on to consider the possible use of Carbon FRP (CFRP) to strengthen and stiffen such timber floors. It is shown that such strengthening and stiffening is entirely feasible, offering the potential for greatly enhanced stiffness, in particular. Further, it is shown that mechanical shear connection between CFRP and timber is best conducted using perpendicular-positioned screws, rather than raked screws.

## 1. Introduction

This paper presents recent research conducted at the University of Bath, UK, into the feasibility of using fibre-reinforced polymer (FRP) materials to complement two distinct forms of timber construction.

Timber gridshells have become a very popular, efficient, sustainable and beautiful structural application of timber [1]. However, given the slender laths involved in this form of construction, there is concern over the durability of timber for this purpose, and Glass FRP (GFRP) laths have been proposed as a possible substitution [2]. This paper considers this possibility. It goes on to look at the possible use of Basalt FRP (BFRP) for the same purpose, from the perspective of its creep characteristics which are crucial in a gridshell system which relies on permanent deformation, during the construction process, and long term loads.

An altogether different form of timber construction is that of joist-and-floorboard. In the UK, there are thousands of historic buildings which use this floor construction, and a sizeable proportion of this building stock now requires upgrade, strengthening and/or stiffening to allow these buildings to be fit for purpose into the future [3]. This paper considers the possible use of Carbon FRP (CFRP) to strengthen and stiffen such timber floors.

## 2. GFRP Gridshells

Gridshell structures are seen as a modern development of shell structures. A shell structure is made of a thin doubly-curved slab that transfers loads through its shell by compressive membrane action instead of by beam-to-beam action by bending and shear [4]. It relies on its double curvature and its in-plane shear properties to achieve its required strength and stiffness to form a load-bearing structure. Gridshells can be created from a flat lattice with rectangular meshes and uniform spacings of the members. As the intersection joints between the members allow in-plane rotation, the lattice therefore has little or no in-plane shear stiffness. This enables the lattice to distort and vary the length of the diagonals in the lattice, and together with the bending of the members, the doubly curved surface of the gridshell can be created.

Loads upon shell structures can be divided into those loads which produce only direct axial compression forces and those which produce bending moments in the laths and, as a result, large deflections; these will be referenced to as funicular loads and disturbing loads respectively. The deformation produced by the disturbing forces changes the shape of the shell from its original funicular form, allowing the direct forces from the funicular loads to produce bending moments, thereby increasing the bending moments caused by the disturbing loads [5]. Characteristic of all compression structures, there exists a critical funicular or axial load at which no resistance to disturbing loads is provided. Owing to the slender nature of the beam elements in gridshells, the possibility of out-of-plane buckling under a relatively low ultimate funicular load is a concern.

Timber gridshells are constructed from thin straight laths. They are often constructed in a flat plane and then jacked into the final doubly curved shell form prior to final fixing at joint positions. Creep and stress relaxation is crucial in this shell forming process in order to prevent overstressing due to the required bending deformation and the possibility of stress rupture due to any remaining locked in stresses.

**Figure 1:**
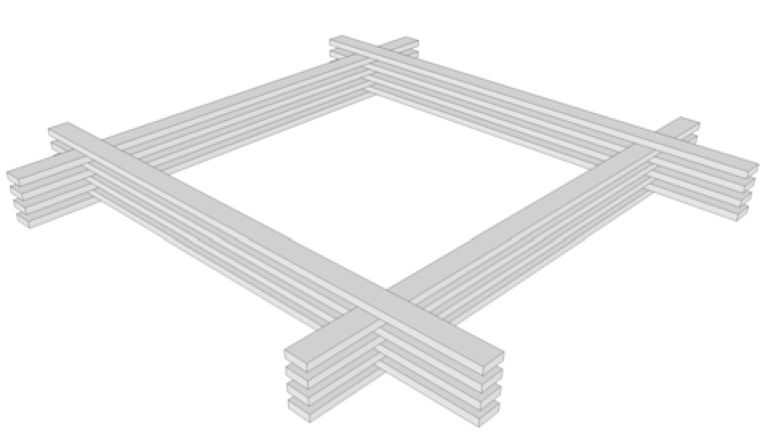
Quadruple layer gridshell element.

To allow deformation of the flat laths into curved shell, the laths must be sufficiently flexible. However, to achieve a gridshell capable of resisting disturbing and funicular loads requires beam elements of a specific cross section. Therefore, when considering large-span gridshells, the size of these beam elements limits the tightness of the curvature that can be developed in the structure when using a single layer of lattice. To overcome this limitation, Happold and Liddell [5] employed a double layer gridshell with the laths being smaller, thereby allowing tighter geometries to be developed, for the Bundesgartenschau in Mannheim, Germany. The installation of shear transfer blocks between the separate lath sections is required to provide an adequate composite action. It is proposed by Harris [1] that multi-layered lattices such as that illustrated in Figure 1 could be utilized to achieve ever-increasing spans.

### 2.1. Introduction of GFRP in Gridshell Structures

The process of introducing curvature to the lattice assembly to form the doubly curved gridshell induces initial bending strains in the elements of the lattice structure. The majority of existing grid shells have employed timber as the structural material owing to its low density and high strain limit (about 2%) in the form of thin flexible laths. The low ultimate strength of timber laths and their relatively low elastic modulus limit the load carrying capacity of these structures and their resistance to disturbing-type loads. Pultruded Glass Fibre Reinforced Polymer (GFRP) materials offer significant improvements in terms of ultimate tensile strength (typically about 350 MPa) and elastic modulus (between 20 and 50 GPa depending on the nature of the fibre reinforcement), combined with a low density of 1,900 kg/m^3^ and a strain limit of 1.5% [6]. For a given geometry of gridshell the buckling load of a composite gridshell will be higher than for the equivalent timber structure. The review of the technical data on the creep behaviour of FRP composites presented by Scott *et al.* [7] in combination with the work of Engindeniz & Zureick [8] highlights the highly durable nature of GFRP components.

**Figure 2:**
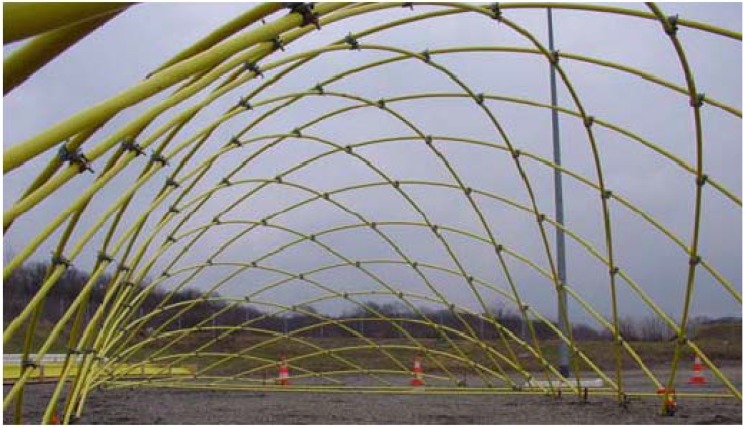
Single layer gridshell constructed of GFRP poles [9].

Figure 2 shows the first single layer gridshell made of GFRP designed and constructed by Douthe *et al.* [9] which is thought to be the first of its kind in the world. Due to the domination of axial loading, unidirectional pultruded GFRP tubes were chosen. Swivel scaffolding elements were employed as node connections as the pultruded profiles were of standard diameter. The structure is 5.80m high with plan dimension of 13.0 m by 13.5 m. It was proposed [9] that steel cables of 5 mm diameter should be used in order to create a stable structure when external loads are applied.

### 2.2. Analytical Assessment of Multi-Layered GFRP Lath Gridshells

Tsui [2] investigated the possibility of creating a traditional lath style gridshell from 20 by 80 mm GFRP laths utilising the flexibility and high strain to failure of the material to achieve highly durable large-span light weight structures. The performance under specific Service Limit State (SLS) conditions and the ultimate funicular load of diagonally braced or un-braced double, triple and quadruple layers of laths were investigated using a non-linear second order FEA model developed using ROBOT Millennium, a commercially available software package for structural analysis.

In order to provide a test bed for the comparison of the different layer gridshells, an elliptical paraboloid of plan area 18.0 by 30.0 m was selected as the set geometry, providing a plan area of 424 m^2^. In-plane shear bracing was supplied in the form of 10mm stainless steel wires running diagonally across the lattice structure. For pultruded GFRP laths of 20 by 80 mm with an assumed long–term longitudinal modulus of 27 kN/mm^2^, the minimum radius of curvature in this set structure was limited to 12 m, developing an initial flexural prestress of 47 N/mm^2^ and a maximum span/rise ratio of 8.

The effects of stress relaxation and creep inherent in GFRP materials [7] have not been considered and the initial prestress is assumed to remain constant with time in this initial analysis. Based on characteristic materials strengths [6] and material partial safety factors applicable to pultruded GFRP, the ultimate allowable stress may be taken as 130 N/mm^2^.

### 2.3. Model Development

**Figure 3:**
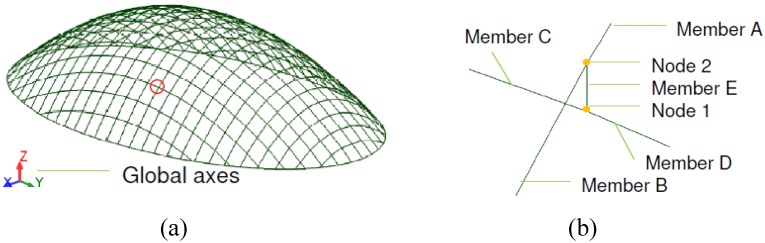
(a) 3-D representation of the FEA model. (b) Arrangement of beam elements at connections.

A set of nodes, based on a 1m × 1m plan grid were defined to represent the doubly-curved ellipsoid surface. To form this doubly curved surface, straight beam elements representative of the double, triple and quadruple lath beams were supplied between the nodes, defining the grid structure of arched beams, as shown in Figure 3(a). Figure 3(b) indicates the nature of the connection between incidental beam elements in the gridshell model. To provide continuity, the nodes between adjoining beams, shown as Member A and Member B in Figure 3(b), are fully fixed such that the beam becomes continuous. The maximum length of any of the individual beam element was approximately 1.3 m.

In-plane rotation was allowed between coincident members using a connection element, normal to the surface, which separates the centriods of pairs of orthogonal curved beams, shown as Member E in Figure 3(b). The node between the connection element and the lower continuous beam which spans the shorter 18m span is fully restrained (Node 1), whilst the node between the connection element and the upper continuous beam, which spans the longer 30 m span (Node 2), allows free rotation about the longitudinal axis of the connection element, thereby allowing relative rotation between the orthogonal elements in the plane of the structure. The connection element was modelled as a steel strut with an elastic modulus of 200 kN/mm^2^ and cross section of 100 mm × 100 mm. The length of this connection element equates to the thickness of one lath (taken as 20mm), representative of the offset produced by splicing multilayer co-incidental beams, as shown in Figure 1. Pinned end constraints were supplied to the ends of the arched beams (at the base of the gridshell) allowing free rotation in all three directions.

As gridshell structures consist of curved slender lath sections under predominately axial forces, they are prone to buckling. Therefore, geometric non-linearity of the structure must be taken into account in order to accurately determine the load at which global buckling occurs. ROBOT Millennium accounts for geometric non-linearity by providing the following modes of analysis.

- Non-linear analysis – takes account of the second-order effects, *i.e.,* change of bending rigidity depending on the longitudinal forces.
- P-delta analysis - takes account of the third-order effects, *i.e.,* additional lateral rigidity and stresses resulting from deformation.

The solution of these non-linear relationships between load and displacement is reached through utilization of the full Newton-Raphson iteration method to introduce incremental changes in the load intensity. Consecutive load increments are only applied to the structure once a state of equilibrium is achieved for the previous increment. The magnitude of unbalanced forces is specified for each step, which allows for monitoring of the structure force-deformation relationships. Enabling geometric non-linearity in the buckling analysis allows for these higher-order effects to be accounted for, improving the convergence of the calculation process for the solution of Eigenvalues that define the buckling modes and, therefore, the collapse load. Since FRP materials are linear elastic to failure, it is not necessary to consider material non-linearity.

 For the structure modelled, the solution to the Eigenvalue problem is generated through the application of the Preconditioned Gradient method using a Multilevel-Aggregation Element-by-Element Iterative Solver (AEBEIS) in combination with Incomplete Cholesky Factorization (ICCF) preconditioning to provide convergence on a buckled shape in less than 100 iterations.

### 2.4. Service Limit State Analysis

**Figure 4:**
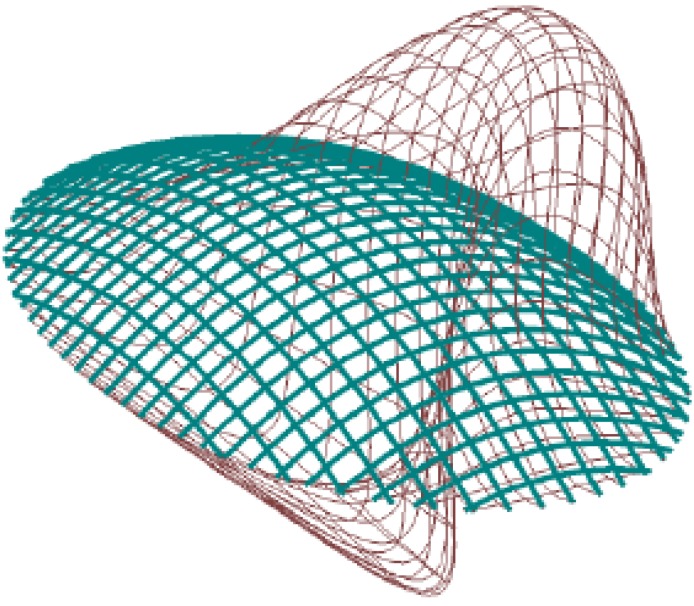
Exaggerated deflected shape under combined snow and wind loading.

To determine the performance of the gridshell structures under in-service conditions, a number of load cases accounting for snow loading of 0.5 kN/m^2^ and wind loading of 1 kN/m^2^ acting either horizontally on the elevation of the structure or vertically as uplift in combination with self-weight of the structure were investigated. Table 1 shows the maximum deflection recorded for each load case for the triple and quadruple layer gridshell calculated using the second order analysis. The position of the maximum deflection was dependent upon the nature of the load case and did not alter between the gridshells. Owing to the high slenderness of the elements, the double layer gridshell failed due to buckling under the combined action of an asymmetrical snow and wind loading, highlighting the influence of disturbing loads on the integrity of gridshell structures. From comparison of the deflection data, in Table 1, it can be concluded that the less stiff triple layer gridshell develops a higher deflection from its original shape for all the load cases as expected. Maximum deflections of 16mm and 10mm for the triple and quadruple layer gridshells were achieved under the worst case combined action of asymmetrical snow and wind loading. Figure 4 gives the exaggerated deflected shape. Although significant, these deflections equate to a maximum deflection to span ratio of 1:1125 and therefore more than amply satisfy the acceptable limits on deflection typically of span/150 for roof members.

**Table 1:** Deflections under SLS loading.

SLS Load cases	Maximum Deflection (mm)		Ratio d_triple_:d_quad_
	Triple layer (d_triple)_	Quadruple layer (d_quad_)	
Snow	1.01	0.86	1.17
Wind A	14.7	9.48	1.55
Wind B	1.58	1.21	1.30
Wind Uplift	0.97	0.607	1.60
**Snow +Wind A**	**16.0**	**10.1**	**1.58**
Snow +Wind B	2.20	1.65	1.33
Snow +Wind Uplift	0.39	0.21	1.86

### 2.5. Ultimate Funicular Load

**Figure 5:**
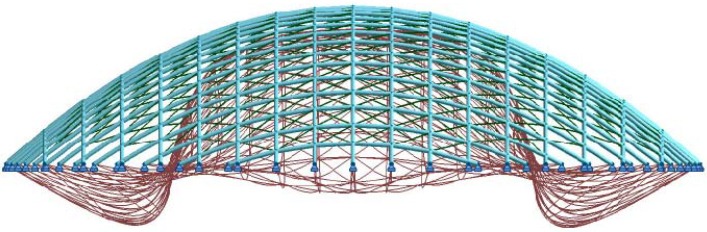
Exaggerated deflected shape under ultimate funicular load.

In order to establish the critical funicular load capacity of each multilayer gridshell, factored dead loads were applied and the calculated maximum deflection reported. Figure 5 illustrates the nature of the deflected shape at the ultimate factored dead load, indicating a symmetrical buckled shape as expected under a critical funicular loading. Table 2 shows the maximum funicular capacity expressed as a factor of the self weight prior to the onset of buckling and the corresponding maximum compressive bending stress for the triple and quadruple GFRP gridshells respectively. The bending stresses reported in Table 2 account for the initial prestress developed during the formation of the double curvature, given as 42 N/mm^2^ to achieve the 12 m radius.

For both cases of braced and un-braced constructions it is clear that the quadruple layer gridshell outperforms the triple layer system by attaining a higher ultimate funicular load. The addition of diagonal bracing increases the maximum funicular loads by a factor of 2.5 and 3.0 times, respectively, indicating that improvements in ultimate funicular capacity can be made with the addition of in-plane shear stiffness. As indicated in Table 2, the maximum stresses recorded in the structures prior to the collapse loads are below the allowable stress defined for the material.

**Table 2:** Ultimate funicular load and maximum stress.

	Triple Layer Self weight (0.20 kN/m^2^)		Quadruple layer Self weight (0.26 kN/m^2^)	
	Maximum Load Factor	Maximum stress (N/mm^2^)	Maximum Load Factor	Maximum stress (N/mm^2^)
Un-braced construction	22	51	30	79
Braced construction	55	79	93	107

### 2.6. Concluding Comments

The initial investigation has shown that triple layer and quadruple layers of GFRP laths can be successfully utilised as structural members for large-span elliptical paraboloid gridshells capable of satisfying specific service limit criteria whilst attaining ultimate funicular capacities in excess of 20 times their own self weight through the development of membrane action. However, one area which needs further investigation is that of creep. Creep and stress relaxation may be beneficial in terms of reducing stresses caused by the gridshell forming process. However, creep rupture due to long term loads and locked in bending and axial stresses may lead to creep rupture occurring at stresses much lower than those allowed under short term load conditions.

## 3. Creep Behaviour of Basalt Fibres

### 3.1. Background

Where FRPs are considered in new construction, such as large span gridshells of the type described above, glass fibres are currently the most commonly considered due to the relatively low cost compared to other fibres, such as aramid and carbon. FRPs using basalt fibres are a possible alternative, but have received relatively little attention up to now. Although the composition of basalt fibres is quite similar to that of glass fibres, there are some compounds which differ, resulting in some differences in performance. Liu *et al.* [10] found basalt fibre composites to have similar Young's modulus, tensile, flexural, shear and compression strength to those of glass fibre composites. However, basalt fibres have been demonstrated to offer better performance than glass fibres, predominantly in more extreme environments, due to the improved behaviour at high temperature, in alkali environments and in the presence of ultra-violet light [11,12]. Therefore, basalt fibres are a genuine alternative to glass fibre, with similar mechanical properties and cost.

However, the behaviour of basalt bars under sustained load (e.g., constant dead load, prestress or locked in bending stresses, as experienced in gridshells described in the previous sections) is not yet fully understood. It is known that sustained loads may lead to sudden unexpected creep (or stress) rupture in advanced fibre composites at loads much lower than the ultimate short term tensile capacity. The more commonly used fibres have fairly well documented creep behaviour. The 500,000 hour (50 year) stress limits, according to Yamaguchi *et al.* [13] are 0.33 f_fu_ for glass, 0.5 f_fu_ for aramid and 0.9 f_fu_ for carbon FRP, where f_fu_ is the short term ultimate strength. These values form the basis of creep rupture guidance in ACI 440.1R-06 [14]. Thus, there are severe restrictions in allowable stresses due to permanent load or prestress conditions. Due to the similarities between basalt and glass, it would seem probable that basalt fibres would have a similar creep rupture behaviour. The following sections examine the creep behaviour of basalt fibres through experimental testing.

### 3.2. Test Specimens

Basalt fibres are usually combined with a matrix to form a fibre-reinforced polymer (FRP). However, tensile creep behaviour of FRP bars has been found to be highly dominated by the fibre behaviour [15], so, for ease of testing, only the fibres themselves have been investigated. It should however be noted that a surrounding matrix might allow fibre stresses to be distributed more evenly between fibres, so testing fibres alone results in a lower bound of the creep behaviour of an FRP material. When testing fibre-type materials, there are three main forms which a specimen can take; a yarn, tow or single fibre. Fallatah *et al.* [16] have suggested that a yarn, rather than a tow, might be appropriate due to the twist and weave of yarn, which would allow distribution of stresses between adjacent fibres to some extent through friction, mimicking that achieved by an FRP. However, this interaction is difficult to correlate directly to an FRP so a parallel filament tow has been used, as it would be in a pultruded FRP product. The basalt tow used in this investigation is BCF 13-2520-KV12 supplied by Magmatech Ltd. and manufactured by Kamenny Vek. The individual fibres have a diameter of 13 micrometers, and the tow consists of 16 strands. Each strand consists of bundles of fibres resulting in a linear mass density of each strand of approximately 150 tex (grams per kilometre). The tensile tests presented in this paper were performed on individual strands. The actual tex of each strand tested varied, so this was measured by weighing 500mm specimen lengths using accurate scales.

### 3.3. Creep Test Procedure

**Figure 6:**
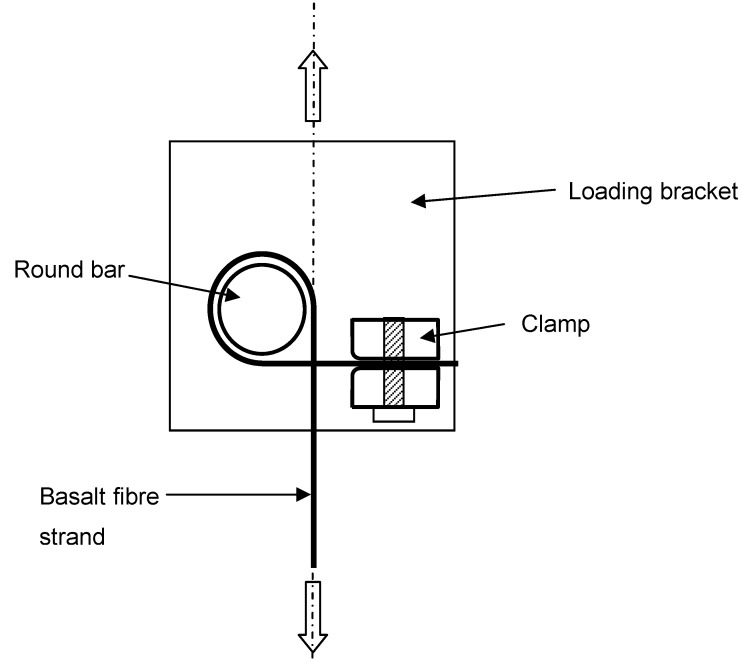
Fibre clamping arrangement.

The specimens were held at each end using specially made clamps. Clamp design is critical to obtaining consistent results, ensuring the clamp itself does not cause fibre damage leading to failure. For the tests carried out in this work, a round bar clamp was used, as shown in Figure 6. The strand was wrapped once around an 8 mm diameter bar before being clamped, allowing friction to reduce the stress in the strand so that the final clamping did not have to carry the full force, thus preventing failure within the clamp itself. A tape was also wrapped around the bar and in the jaws of the clamp so that any surface irregularities did not affect the fibres.

All testing was carried out in a room temperature environment. A laboratory with no opening windows or external doors, and few users, was used so as to maintain a reasonably constant environment. The temperature and humidity were recorded throughout the tests to ensure any significant changes in conditions could be accounted for.

Two sets of tests were carried out in order to ascertain the creep rupture behaviour. Firstly it was necessary to establish the absolute breaking load (ABL) of the strand. Secondly, creep tests were performed by applying a constant percentage of this ABL to several specimens. The elongation of the strand was measured throughout the test. Due to the testing method, the initial strain upon loading was calculated using the elastic modulus of the fibres, which was established from additional short term non-destructive tests.

The ABL tests were carried out using an Avery Type 6601 tensile testing machine, which uses a hinged lever-arm that provides a 40× multiplication of loads and displacements between the point of load application and the specimen. The specimen was fixed into the machine using round bar clamps at either end and the load applied by steadily releasing lead shot into a container on the end of the lever arm. Loading stopped when the strand snapped, and the breaking load was then calculated. A total of ten specimens were tested in this way in order to obtain a mean strength.

The creep rupture tests were carried out on a separate test rig to allow multiple specimens to be tested at the same time. The samples were again held using the same round bar clamps but, in this case, were loaded using hung weights. Elongation of the strand was measured by means of a dial gauge measuring the vertical displacement of the weights, allowing strain in the specimen to be calculated. For these creep rupture tests, three specimens were tested at each of four loads; 82%, 76%, 72% and 65% of ABL.

### 3.4. Creep Test Results and Discussion

**Figure 7:**
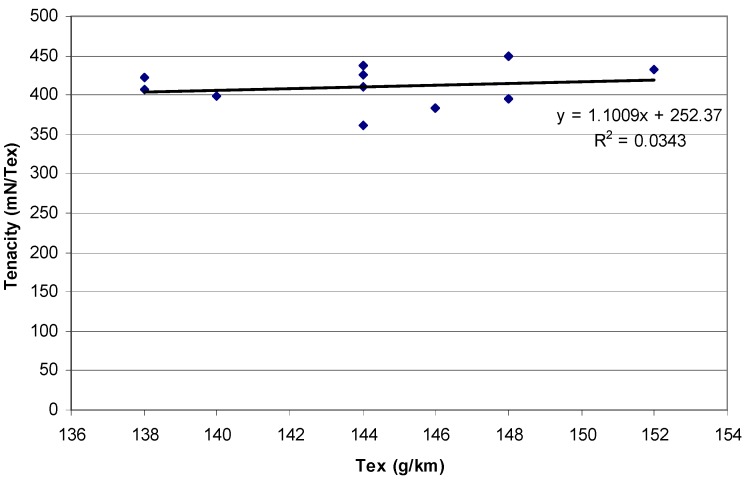
Tenacity (ABL/tex) against strand linear density (tex).

Figure 7 shows the results of the short-term ABL tests, plotted as tenacity (ABL per tex) versus tex, for each sample. The tenacity ranges from 361 to 449 mN/tex with an average of 411 mN/tex. A best fit linear regression line is fitted through the data and the low determination coefficient indicates high scatter about the regression line. There appears to be no correlation between the independent and dependent variables. Average values are nevertheless used although there is no real justification for this. The average tex of the specimens tested was 144 g/km, so the average ABL is 59.2 N. To ensure consistency for the creep rupture tests, specimens with a tex less than 142 or greater than 148 g/km were rejected.

**Figure 8:**
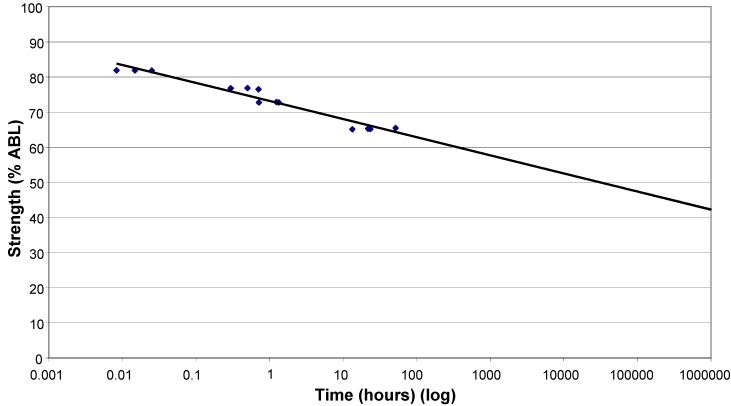
Creep rupture test results.

The results of the creep rupture tests are shown in Figure 8, using a log time scale. Chambers and Burgoyne [17] suggest that the linear relationship between stress and the log of lifetime is consistent with various theories for stress rupture and it is therefore justified theoretically to extrapolate the best fit line. There is a clear increase in the length of time which the basalt strands can withstand a constant load as that load reduces. Extrapolating to 500,000 hours, the load which can be carried appears to be 43% of ABL. This is higher than the 33% found for glass fibre [13], but is based on very limited experimental data, so the confidence in this as an absolute value is low, particularly given the scatter of results and the degree of extrapolation.

**Figure 9:**
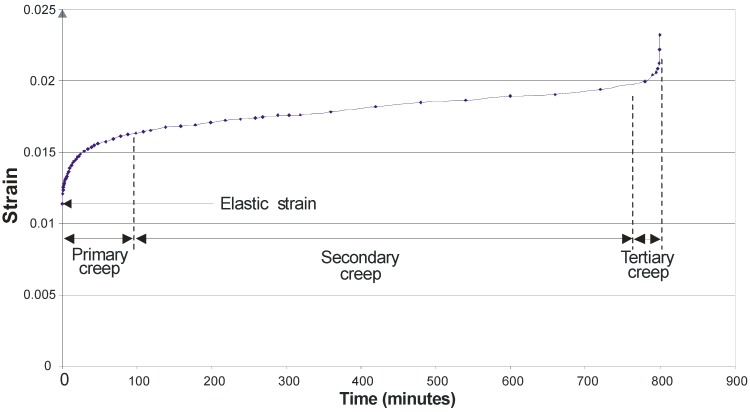
Creep strain curve (65% ABL).

**Figure 10:**
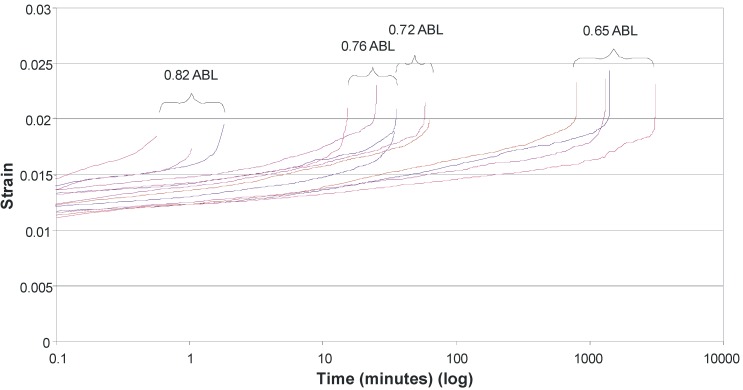
Creep strain curves – all tests on log time scale.

**Figure 11:**
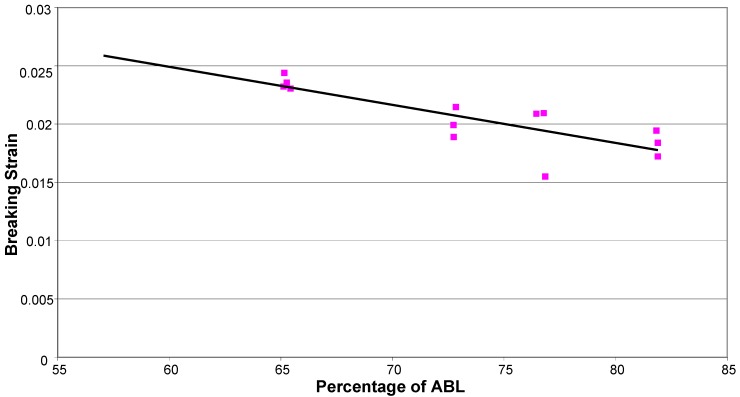
Breaking strain plotted against %ABL.

Examining the time-dependent creep behaviour of the strands throughout the creep process reveals behaviour similar to that observed for aramid tow [17]. Figure 9, for a specimen tested at 65% of the ABL, exemplifies the behaviour observed for all specimens. Initially as the load is applied the sample undergoes elastic strain. There are then primary, secondary and tertiary creep stages. The fibres undergo primary creep as they bed in, and initial effects take place. Secondary creep is a period of low creep rate which remains fairly constant over an extended period of time. As the cross sectional area decreases, due to both Poisson ratio effects and individual fibre rupture, the stresses steadily increase and tertiary creep occurs, which precedes failure. If the strain is plotted on a log time scale for all specimens (Figure 10) it can be seen that, broadly speaking, as time to failure increases, so the total strain to failure increases, despite the smaller loads applied for the longer time periods. This is the reverse of what has been observed for aramid fibres [17]. Plotting failure strain against %ABL (Figure 11) demonstrates this counter-intuitive relationship. This is also at odds with research by Fujii *et al.* [18] which suggests that creep rupture failure strain is constant and independent of the applied stress for glass FRP bars in tension, but this may be due to the influence of the matrix. It therefore seems that a strain limit is not an appropriate creep rupture indicator for basalt fibres, but rather a stress limit.

### 3.5. Concluding Remarks

The important observation from the creep rupture experimental data is that there is a clear and significant reduction in load capacity of basalt fibres under long term loads, which is of a similar order to that of glass fibres. Preliminary results suggest that the permanent load limit to allow a 50 year life is around 40–45% of the short term strength of basalt fibres. Therefore, once safety factors have been added, the creep rupture behaviour of basalt bars severely limits the use of basalt under permanent load conditions. It would be necessary to carry out further long term testing and/or accelerated tests to accurately establish a safe long term permanent working load. Furthermore, it may be necessary to test basalt FRP elements where the interaction of the fibres with a resin matrix can be taken into account in order to directly compare with results of glass FRP, and also to be able to determine compression-dominated creep behaviour, as would be encountered in gridshells.

## 4. CFRP Strengthening and Stiffening of Timber Flooring

### 4.1. Background

Recently there has been growing concern about how the flooring systems of historical buildings will stand up to the increasingly larger loads that are being imposed on them. This refers to the profound refurbishment of the UK’s infrastructure which is ongoing, and to the growing tourism industry in the UK which means that stately homes have increasing numbers of visitors to their sites. In both situations, timber floors which are of insufficient strength and stiffness need to be structurally retrofitted. This retrofit must occur on the top of the floor as access to the joists through historic lower-storey ceilings is not possible. The retrofit must also be reversible, a fundamental principle in historical conservation work.

One issue that is created by reinforcing the top of a flooring system is that internal dimensions of the rooms will be affected. For this reason a thin overlay is essential so that the dimension change is minimal. FRP composites need only a small cross sectional area to provide a significant increase in strength and stiffness to a structural system, even if the FRP is acting in compression only. This relationship is achieved through shear connection between the FRP topping and the timber joists. There has been widespread research into timber-FRP connections with a focus on adhesives to create the composite action [19]. Adhesives are extremely efficient in transferring load but are inappropriate for interventions of this kind since the retrofit system cannot be easily removed to return a building to its original state, as is desirable for conservation work. Alternative mechanical-fastening methods are necessary, relying on nails, bolts or screws. The impact of these connectors is less serious than that of adhesives, but it is crucial that the number of such connectors is minimised for the essential structural gains which are needed. In the present project, bolts are not a feasible connector, and nails have installation (damage to CFRP) and deconstruction issues (many are needed, leading to reversibility issues) associated with them. The use of screws becomes the only feasible connection technique in order to achieve as far as possible a composite action between FRP and timber. Note also that mechanical fastening carries with it no chemical-adhesive drawbacks, such as curing times, fumes or pot-life issues.

It would not be feasible to overlay an entire floor with a thin sheet of FRP, but it is indeed entirely feasible to place FRP strips of, say, 50mm width and a few millimetres thickness along the lines of one-way-spanning joists, thereby adequately stiffening and strengthening the joists locally, practically and quickly. Clearly, the strips will need to be axially very stiff in order to contribute significantly to overall stiffness and strength, so CFRP has been chosen.

It is known that under compression, local effects in CFRP are crucial for overall strength [20]. Thus, the mechanical shear connection efficiency and effectiveness are critical here. Clearly, the CFRP will need to have multi-directional fibres in order to diffuse local effects at connections, as found by Lamanna *et al.* [21] during their mechanical fastening of CFRP strips to concrete structures.

### 4.2. Long-Term Effects and Composite Action

The long term behaviour of FRP bonded onto substrates is still not fully understood because of its relatively recent introduction into the construction industry [22]. However, much of the work that has been carried out has shown that the problematic effects are generally issues to do with the adhesive layer [23]. What has not yet been satisfactorily looked into is the long term behaviour of FRP associated with using mechanical connectors. There is, of course, also the issue of creep in CFRP materials under compression, although CFRP behaves rather well in this regard [24].

Clearly, it is desirable to have as stiff a connection as possible between FRP and timber, to reduce slip and maximise composite action [25]. Frangi & Fontana [26], Whitworth [27] and Neve [28] conducted research into a thin concrete topping instead of using FRP, and all discovered that profound composite action is possible if the connection is designed appropriately.

**Figure 12:**
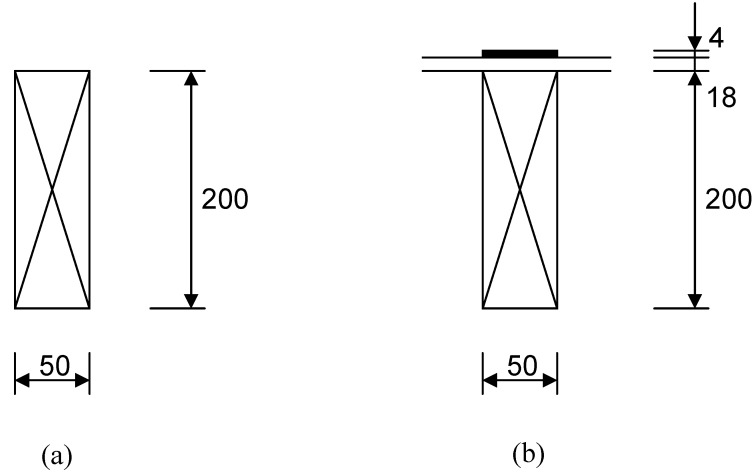
(a) Joist only. (b) Joist with 4mm-thick CFRP topping.

Assuming full composite behaviour, it can easily be shown [3] that the bending stiffness of a timber joist of typical dimensions in the UK of 200 mm in depth and 50mm in width (Figure 12 (a)) is roughly doubled when a 4 mm-thick, 50 mm wide unidirectional CFRP strip is attached rigidly to the joist using screws which go through an 18mm-thick floorboard (Figure 12 (b)). This extraordinary increase in stiffness, and hence strength, of the system using this simple technique is therefore clear, and it becomes a question of how to achieve the assumed fully-composite behaviour.

**Figure 13:**
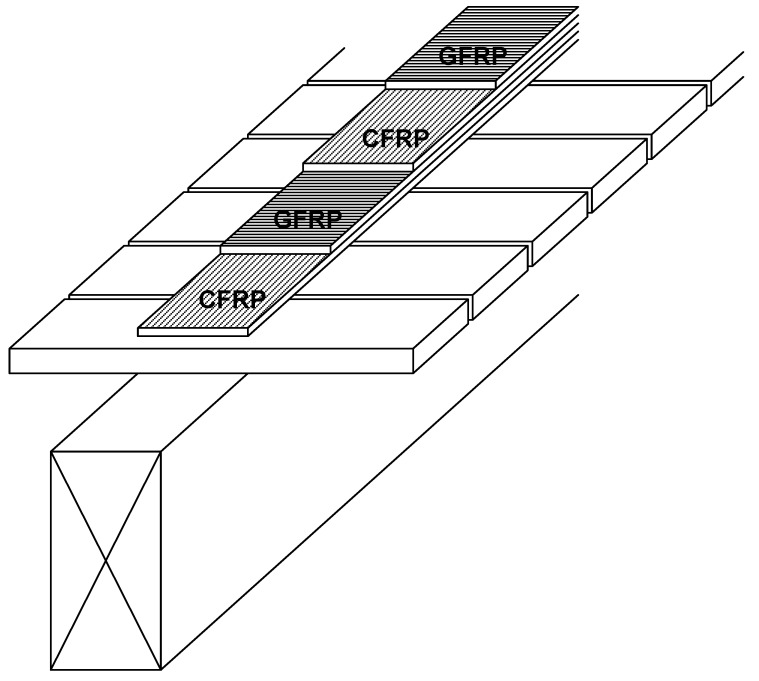
Layering-up of the FRP topping using two plates of 2 mm-thick CFRP and two plates of 3 mm-thick GFRP.

### 4.3. FRP and Timber Materials

Bidirectional CFRP is an expensive material and not widely produced. Therefore, a hybrid CFRP-GFRP was chosen to be used here. The CFRP provides the longitudinal strength and stiffness, while the GFRP provides the transverse integrity for local connections to be possible. The behaviour of CFRP-GFRP material has been studied by Bader and Manders [29] and this work guided the design of the interaction between these two FRP materials here. It was decided to use two 2 mm-thick CFRP unidirectional plates in the longitudinal direction (of stiffness 120 GPa), sandwiching two 3 mm-thick GFRP unidirectional plates in the transverse direction (of stiffness 25 GPa), as shown in Figure 13. The adhesive used to create this sandwich arrangement was Sikadur 30.

**Figure 14:**
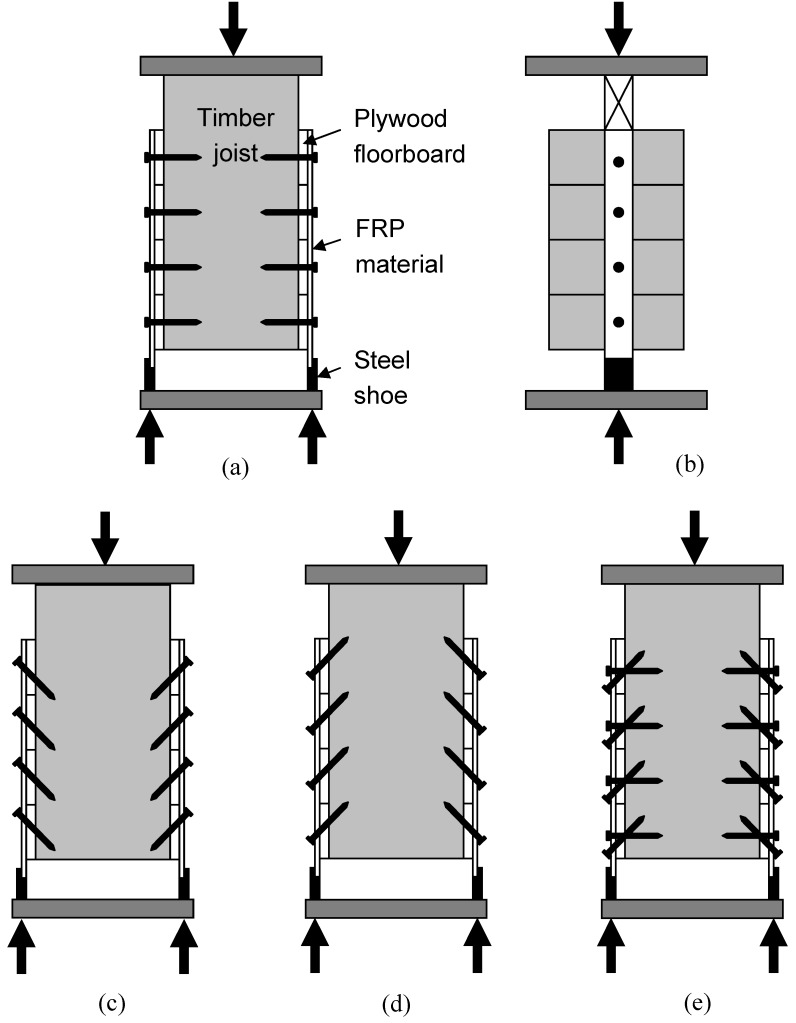
(a) Front view of Specimen 1. (b) Side view of Specimen 1. (c) Specimen 2. (d) Specimen 3 (e) Specimen 4.

Douglas Fir of grade C16 and dimensions 47 mm × 225 mm was chosen for the joists, with 18mm-thick plywood boarding used between the FRP and joists. Joist timber for each of the push off specimens described below was cut from the same length of timber to minimise non-uniformity of material properties between tests. The steel screws chosen were about 88 mm long and nominally 5mm in diameter, and roughly 1300 MPa in direct measured tensile strength. The FRP was pre-drilled to receive these 5 mm screws in order to minimise damage to the composite, but the timber was not predrilled in any way since the screws were self-tapping.

### 4.4. Push-off Testing

**Figure 15:**
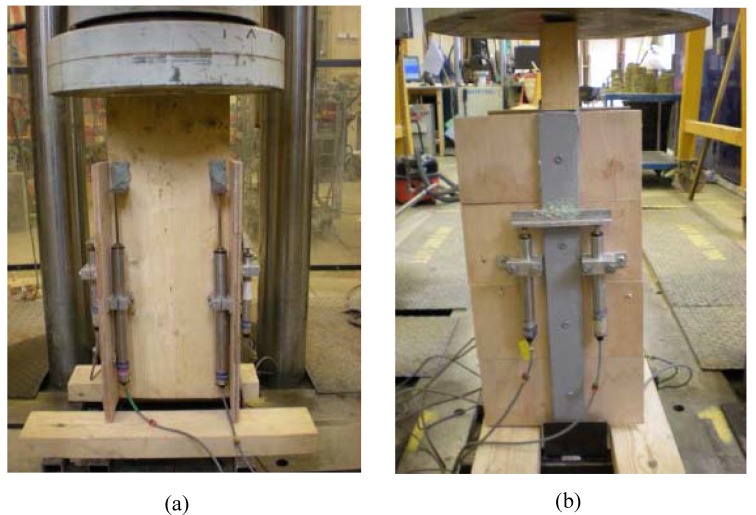
(a) Front view of specimen in test rig. (b) Side view of specimen in test rig.

The proposed FRP-timber system relies on composite behaviour, so push-off testing was chosen as being most appropriate to study this. Figure 14 shows the test configuration of each of the four symmetric push-off specimens considered, while Figure 15 shows the typical test set-up arrangement in a 2000kN-capacity rig. Displacement transducers are clearly visible, with eight having been used in each test arrangement to measure relative slips between FRP and plywood, and between plywood and timber joist.

Test Specimen 1 contained perpendicular-positioned screws. Test Specimen 2 contained raked screws at 45°, placing them in tension for the push off test. Test Specimen 3 contained similarly-raked screws, but placing them in compression. Test Specimen 4 combined the connector geometry of samples 1 and 3. All specimens were loaded in shear at a rate of 5mm/minute.

### 4.5. Results and Discussion

**Figure 16:**
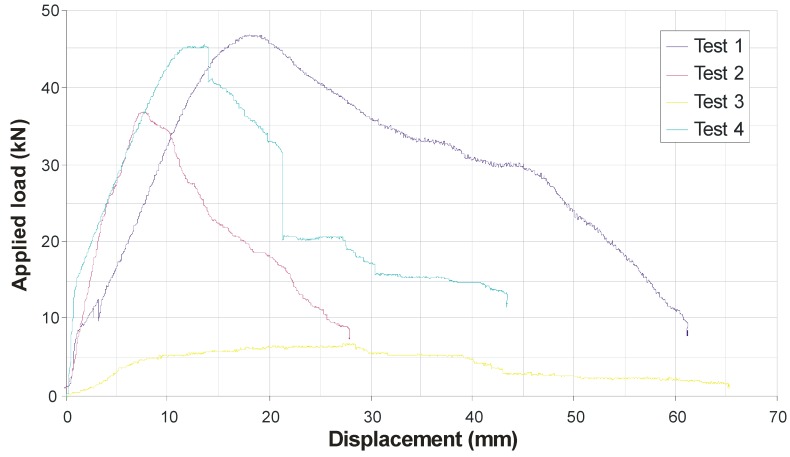
Load-Displacement plots for all four specimens.

Figure 16 shows the load-displacement plots for all four specimens. Specimens 1 and 2 exhibited failure in the timber (screw pull-out), while Specimens 3 and 4 (containing raked screws in compression) exhibited bearing failure in the FRP. Specimens 1 and 4 (containing perpendicular-positioned screws) clearly fared better than Specimens 2 and 3. Further, Specimen 3 (with only raked screws in compression) clearly failed to improve capacity and stiffness to the same extent as the other specimens. It is evident that screws should not be raked in compression in such systems. Given that load reversal is always possible in structures, it follows that raked screws should probably be avoided.

It is possible to consider a limiting upper-bound shear capacity on the system by determining the shear capacity of the screws alone along the shear planes. According to Tresca’s Yield Criterion, the yielding capacity of each screw in pure shear will be half its uniaxial tensile yield strength, which itself was found to be about 535 MPa if one assumed each screw was uniformly 5 mm in diameter along its length. By summing the upper-bound shear resistance from all eight screws under full shear-yielding conditions, it may easily be shown that the capacity of the specimens would be about 42 kN [3]. This compares rather favourably with the peak capacities encountered in specimens 1 and 4, which were around 45 to 47 kN. This implies that fully-composite behaviour has been achieved in these two specimens, as the limiting capacity of the shear strength of the screws appears to have been reached. Indeed, after failure of the specimens, it was noticed that the screws in specimens 1 and 4 were all significantly deformed.

### 4.6. Concluding Comments

It has been demonstrated that using screws to create the shear connection between structural timber floor joists and an FRP topping plate is a suitable method, with full composite behavior being achieved when perpendicular-positioned screws are used. It seems possible to double the bending stiffness of timber flooring systems in this way, whilst preserving the fabric of the building for the future and ensuring minimal intervention geometrically on the internal space in the building.

Clearly, these pilot tests are merely the start, and all they demonstrate at this stage is that this retrofit technique deserves further investigation.

## 5. Conclusions

This paper has demonstrated that FRP materials can be used appropriately to form extraordinary new-build structures in a durable manner. It has also shown that existing timber flooring systems can be upgraded significantly in a structural sense by adding suitable FRP systems.
